# Reliability of on-line visual feedback influences learning of continuous motor task of healthy young adults

**DOI:** 10.3389/fpsyg.2023.1234010

**Published:** 2023-10-06

**Authors:** Marie Bernardo, Yannick Blandin, Géry Casiez, Cécile R. Scotto

**Affiliations:** ^1^Centre de Recherche sur la Cognition et l’Apprentissage, Université de Poitiers, Université François Rabelais de Tours, Poitiers, France; ^2^Univ. Lille, CNRS, Inria, Centrale Lille, UMR 9189 CRIStAL, Lille, France; ^3^Institut Universitaire de France (IUF), Paris, France

**Keywords:** cue reliability, sensorimotor coding, sensorimotor learning, visual feedback, motor sequence

## Abstract

A continuous task was used to determine how the reliability of on-line visual feedback during acquisition impacts motor learning. Participants performed a right hand pointing task of a repeated sequence with a visual cursor that was either reliable, moderately unreliable, or largely unreliable. Delayed retention tests were administered 24 h later, as well as intermanual transfer tests (performed with the left hand). A visuospatial transfer test was performed with the same targets’ sequence (same visuospatial configuration) while a motor transfer test was performed with the visual mirror of the targets’ sequence (same motor patterns). Results showed that pointing was slower and long-term learning disrupted in the largely unreliable visual cursor condition, compared with the reliable and moderately unreliable conditions. Also, analysis of transfers revealed classically better performance on visuospatial transfer than on motor transfer for the reliable condition. However, here we first show that such difference disappears when the cursor was moderately or largely unreliable. Interestingly, these results indicated a difference in the type of sequence coding, depending on the reliability of the on-line visual feedback. This recourse to mixed coding opens up interesting perspectives, as it is known to promote better learning of motor sequences.

## Introduction

During motor learning, humans rely mainly on visual information to produce accurate and stable behaviors (for a review, see Wolpert et al., 2011). According to the specificity of practice hypothesis (Tremblay and Proteau, 1998), although visual feedback initially helps to improve performance, it can lead to forms of dependency. As a result, when this feedback is removed, performance in delayed retention collapses. Studies have shown that in addition to movement coding based on visual cues (i.e., visuospatial coding), participants engage either in motor coding (Hikosaka et al., 1999, 2002) based on proprioceptive cues, or in a combination of the two (Kovacs et al., 2009). Moreover, in the case of unreliable visual feedback during the learning of a discrete task, they rely on other sensory sources (Körding and Wolpert, 2004; Berniker and Kording, 2008; Hewitson et al., 2018), as predicted by the Bayesian approach to multisensory integration (Ernst and Banks, 2002). Most of the work on the motor learning process has involved the use of discrete tasks, even though most of our daily actions are sequential (e.g., including several sub-tasks such as walking or brushing teeth; Hikosaka et al., 1999). In the present study, we therefore assessed the impact of on-line visual feedback reliability on the learning of a motor sequence.

Sensorimotor learning requires individuals to identify the source of information most likely to ensure maximum success on the task (*specificity of practice hypothesis*; Tremblay and Proteau, 1998). They do this early in practice, and rely on that information thereafter, to the detriment of other sensory sources. If a motor task requires high visuospatial accuracy, for example, visual feedback is rapidly established as the dominant source (Tremblay and Proteau, 1998). However, if this information subsequently becomes unavailable, the planning and control of movement will be disrupted, resulting in major aiming errors. Most of the studies testing the specificity of practice hypothesis have focused on the manipulation of task constraints (e.g., practice conditions or target sizes), mainly for discrete tasks (e.g., Robin et al., 2004; Proteau, 2005). However, Blandin et al. (2008) focused on the learning of a continuous arm flexion-extension sequence, showing that whereas the on-line visual feedback of the effector’s position was initially beneficial (i.e., during acquisition), it became detrimental during retention and transfer, if participants no longer had access to it. This was an important result, as it validated the specificity of practice hypothesis (Tremblay and Proteau, 1998) for sequential tasks and confirmed the evolving role of feedback across the different stages of learning (for a review, see Wolpert et al., 2011).

Moreover, the representation of motor skills relies on distinct and independent coordinates or coding systems (Coello et al., 1996; Criscimagna-Hemminger et al., 2003; Lange et al., 2004). Each coding system contributes to movement production and can produce specific learning and transfer capacities. According to Hikosaka et al. (1999, 2002), the learning of a motor sequence is encoded in a neural-network schema in two ways (i.e., visuospatial and motor coding). The spatial locations of the effector and the targets to be reached are coded in visuospatial coordinates. This coding, the dominant representation early in practice, is explicit (i.e., humans consciously use relevant visual information such as the positions of their limbs and the stimuli), and effector-independent (i.e., not specific to an effector). Additional practice leads to motor coding: motor patterns (i.e., activation of agonist/antagonist muscle) generate muscle activations and joint configurations specific to the effector producing the movement. Several studies have confirmed this initial dominance of visuospatial coding (e.g., Park and Shea, 2002, 2005; Kovacs et al., 2009; Boutin et al., 2010, 2012), and the relative underuse of motor coding (e.g., Kovacs et al., 2009; Boutin et al., 2010, 2012) through intermanual transfer tasks. During these classical transfer tasks, participants perform the sequence with their non-dominant hand and either the same targets’ sequence to point (which maintains the visuospatial coding; visuospatial transfer test, TVS) – or the mirror of this targets’ sequence (which maintains the motor coding; motor transfer test, TM). A higher performance in TVS would imply a higher visuospatial coding while a higher performance in TM would imply a higher motor coding (e.g., Park and Shea, 2002, 2005; Kovacs et al., 2009; Boutin et al., 2010, 2012).

The reliability of visual information has recently been identified as a potential factor influencing coding processes and, more particularly, promoting the implementation of motor coding (Robin et al., 2004; Hewitson et al., 2018): when visual information is unreliable, participants rely on proprioceptive information and undertake more motor coding. This hypothesis is based on the work of Körding and Wolpert (2004) who showed that participants exposed to visuomotor bias adapted their pointing movements relative to the level of uncertainty of the sensory feedback. In this study the feedback reliability was manipulated by providing the cursor position through the presentation of multiples dots distributed as a two-dimensional gaussian depicting a cloud of dots. Participants learned the sensory feedback likelihood over practice trials (Sato and Kording, 2014). The weight assigned to each sensory modality therefore depends on its relative reliability for the participant (Ernst and Banks, 2002).

In the present study, we used a continuous dynamic arm movement task to examine motor sequence learning according to the reliability of the on-line visual feedback. We specifically assessed whether coding of the task depends on this reliability, which was not already shown to our knowledge. We choose to manipulate the feedback reliability with a paradigm similar to Körding and Wolpert (2004) and Hewitson et al. (2018) which depicted a cloud of dots. Therefore here three conditions of reliability were tested: reliable (σ0), Moderately unreliable (σM), and Largely unreliable (σL). We predicted that the more unreliable the visual cursor, the harder the sequence would be to learn, as shown for discrete tasks. Because many learning experiments reported that unfavorable practice conditions usually favor learning (Sigrist et al., 2013 for a review), we first had to assess the effect of feedback reliability on learning. Moreover, we predicted a more efficient motor coding would take place in the two unreliable conditions than in the reliable condition. Indeed, as participants who could not trust the visual information would rely more on proprioceptive information and favor motor coding.

## Methods

### Participants

We recruited 48 adults (mean age = 19.19 years, *SD* = 1.14; 16 women and 32 men) among students at Université de Poitiers, who received a course credit for taking part. All participants stated that they were right-handed. They each signed an informed consent form prior to the experiment, which was approved by the local ethics committee (no. 201965). All participants stated that they had no history of neurological or sensorimotor disorders, had normal or corrected-to-normal vision, and had not consumed energizing substances in the 24 h preceding either phase of the experiment. Participants were randomly assigned to one of three groups: σ0 (reliable cursor with *Zero* uncertainty), σM (*Moderately* unreliable cursor), or σL (*Largely* unreliable cursor; see *Task, Groups and Procedures* for details).

An *a priori* power analysis for F-tests (compute required sample size - given α, power, and effect size) conducted with G*power 3.1 (Faul et al., 2007) using the power of 0.95 and the effect size of ηp^2^ = 0.84 from the between-within-subject interaction of the Boutin and Blandin (2010) experiment indicated that the inclusion of 15 participants in each group would be sufficient. However, the counterbalancing of retention and transfer tests on Day 2 with sub-groups required that the number of participants was a multiple of 4. Therefore, we chose to include 16 participants per group: σ0 (2 women, 14 men; mean age = 18.81 years, *SD =* 0.75), σM (7 women, 9 men; mean age = 20.06 years, *SD* = 1.34), and σL (7 women, 9 men; mean age = 18.69 years, *SD* = 0.70). A one-way between-subject ANOVA was conducted on the initial control block (i.e., R0) to assess a possible gender effect due to umbalanced gender between groups (σ0 = 2 women vs. 7 women in σM and σL) and failed to reach significance [*F*(2,45) = 1.39; *p* = 0.26].

#### Apparatus

The task was controlled by a Dell computer [Intel(R) Xeon(R) W-2123 CPU @3.60GHz with Windows 10 professional system] with a high-definition screen (Acer ROG PG278QR, 2,560 × 1,440 pixels, refreshed at 165 Hz, connected to an NVIDIA GeForce GTX 1080 graphic card) and a high-definition graphics tablet (WACOM Intuos4 XL 1240-d version 2.0, resolution: 5080 lines per inch – 0.005 mm per point, sensitive area: 493 × 304 mm, refreshed at 200 Hz). A digital stylus allowed participants to navigate the screen through the tablet. Data from the stylus were processed by a custom-built application written in C++ using Qt. We used absolute mapping between the tablet and screen, with a gain value of 1 (i.e., what was seen on the screen corresponded to what was done on the tablet). The center of the screen corresponded to the center of the tablet. The cursor was only displayed on the screen when the stylus was in contact with the tablet.

### Task, groups, and procedures

**Figure 1:**
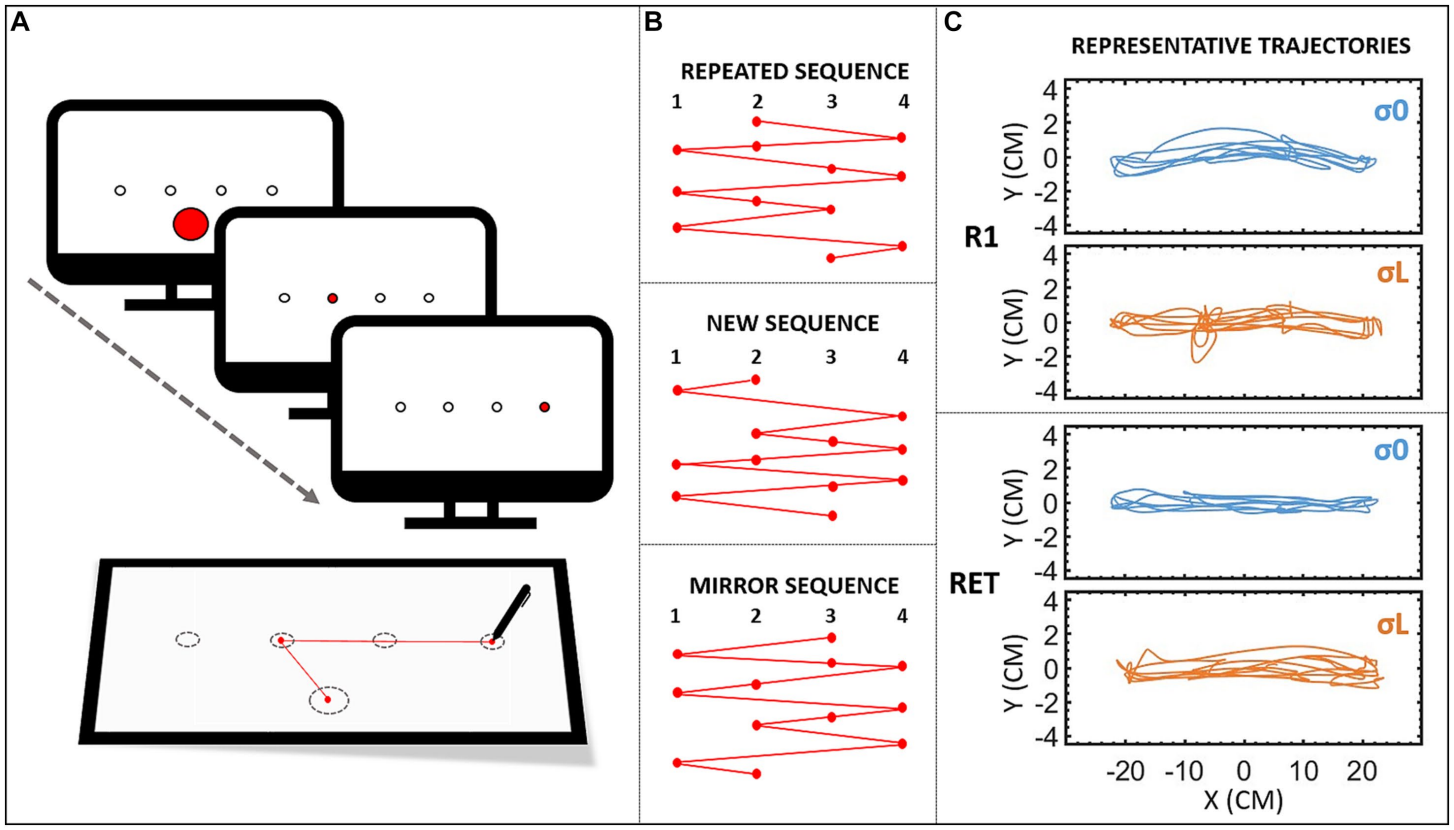
**(A)** Set-up. To launch the block, participants had to point to a centered calibration target at the bottom of the screen. This target then disappeared, and participants had to reach the targets as soon as they lit up, without lifting the stylus from the tablet. **(B)** Sequences. Design of the three sequences. The repeated sequence was the sequence participants had to learn, and was used in all the repeated blocks (*R*_0_–*R*_20_), as well as the retention blocks (RET_C_ and RET), and the visuospatial transfer block (TVS). The new sequence was different from the repeated sequence, but with the same characteristics, and was used in the pre- (*N*_1_) and post- (*N*_2_) tests. The mirror sequence (mirror of the repeated sequence) was used in the motor transfer block (TM). **(C)** Trajectories (X vs. Y positions in cm) of a representative participant from groups σ0 (reliable cursor) and σL (Largely unreliable cursor) during the last targets’ sequence of 12 elements of the first repeated bloc (R1) and the retention block (RET).

Four targets (⌀ = 1 cm each) were displayed on the screen, aligned horizontally at −21, −7, 7, and 21 cm from the center (Figure 1A). To facilitate the procedure’s understanding, we named the targets according to their position starting from the left (i.e., 1, 2, 3, and 4; Figure 1A; participants were not aware of this labeling). When a target was active (i.e., when participants had to point to it), it turned red, otherwise, only their circular outlines remained visible. Participants had to point to a centered *calibration target* (⌀ = 6 cm) at the bottom of the screen to start each block of trials. Once they had done so, this target disappeared.

Prior to the experiment, participants were asked to adjust the height and position of their chair, so their right hand was at desk height and approximatively in the center of the tablet. The task consisted in making arm-pointing movements toward the targets, which were displayed in an ordered sequence. A typical practice session began with participants being told to move the stylus to the calibration target (see Figure 1A). The first target then turned red, and participants had to move the stylus across the tablet as quickly as possible, in order to validate it by crossing the circle’s perimeter: participants did not have to stop the cursor in the target. As soon as the target was reached, it lost its red color and the next target turned red, and so on and so forth, until the sequence of 12 elements (i.e., targets; Figure 1B) was complete. This sequence was repeated 10 times in each block. When a block was finished (i.e., 120 successive targets), participants could take a break (i.e., at least 5 s) before beginning the next block. During the acquisition phase, the movement time (MT) of the full block of trials (i.e., 120 repetitions) was displayed on the screen at the end of each block, so that participants could use this feedback to improve their performance. Pointing movements were performed with the right hand except for the two transfer tests (described below). Participants were instructed not to lift the stylus from the tablet, otherwise the cursor would disappear. Representative trajectories of sequence’s pointing are depicted in Figure 1C.

Three different sequences were used during the experiment, in accordance with Boutin et al. (2014). They were each composed of 12 elements (Figure 1B). The *repeated sequence* (targets: 2 4 2 1 3 4 1 2 3 1 4 3) was the sequence to learn. The *new sequence* (targets: 2 1 4 2 3 4 2 1 4 3 1 3) was an unpracticed sequence used for the pre- and posttests. Finally, the *mirror sequence* (targets: 3 1 3 4 2 1 4 3 2 4 1 2) was used for the motor transfer block.

**Figure 2:**
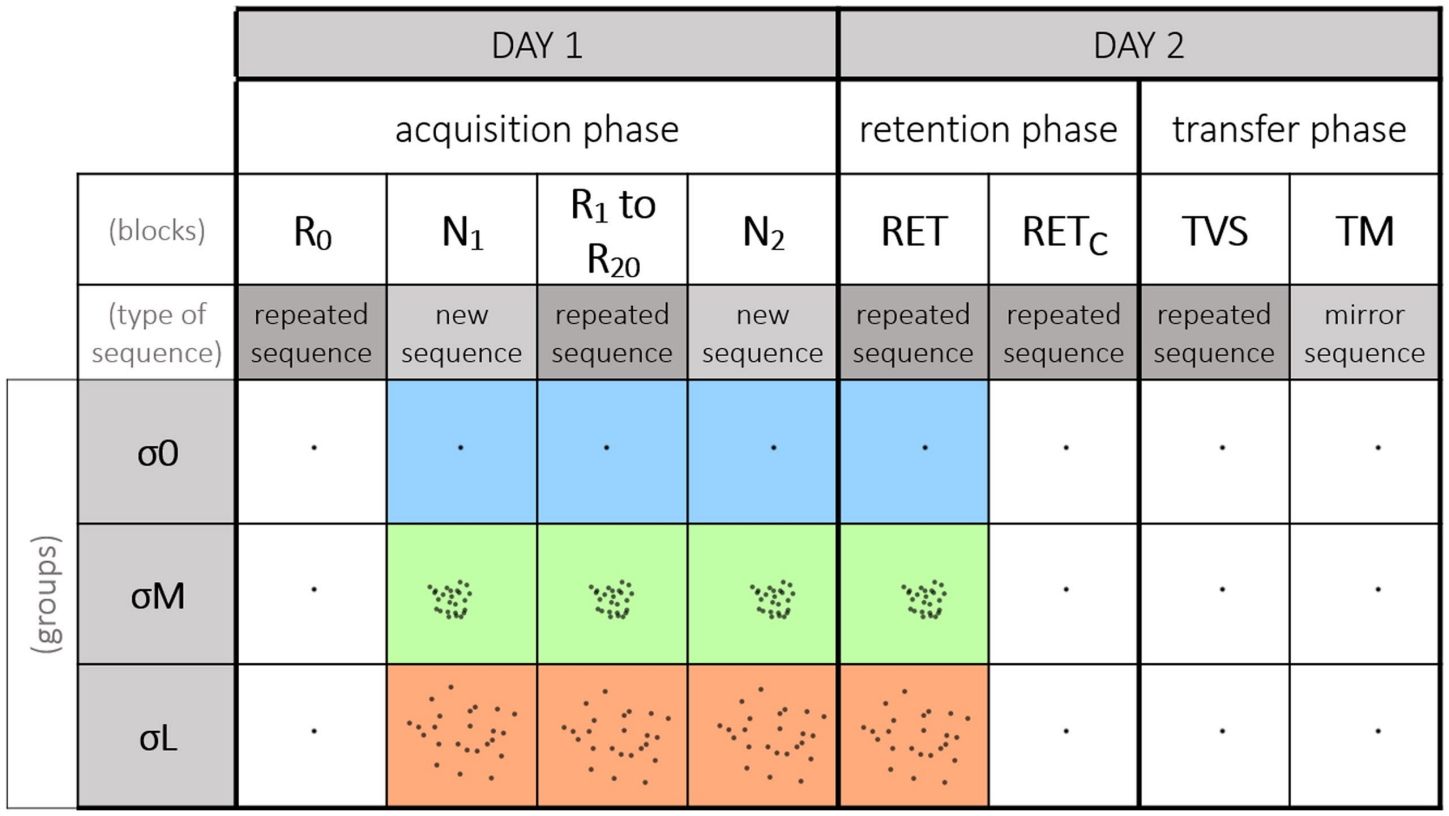
Experimental procedure. The figure depicts the blocks order relative to the cursor reliability. A block corresponds to 10 repetitions of the 12 elements sequence. On Day 1, participants performed the acquisition phase, starting with a block featuring the repeated sequence and a reliable cursor (*R*_0_). For the rest of the acquisition phase, participants used their group’s cursor. *R*_0_ was followed by a block featuring the new sequence (*N*_1_). Participants then performed 20 blocks featuring the repeated sequence (*R*_1_–*R*_20_). Lastly, they performed a second block with the new sequence (*N*_2_). On Day 2, all the participants performed one retention block with their group’s cursor (RET) and another retention block with the reliable cursor (RET_C_) which were counterbalanced across participants. Note that for the σ0 group both RET and RETc were performed with the reliable cursor. Participants also performed two transfer tests with the reliable cursor: one visuospatial transfer (TVS) and one motor transfer (TM), also counterbalanced across participants.

Three groups were defined according to the reliability of the visual cursor they used during the acquisition phase. The reliability of the cursor was manipulated through the size and density of a cloud of dots (Körding and Wolpert, 2004; Figure 2). In the *reliable condition* (σ0), the cursor was a single black dot (⌀ = 1 mm). In the *moderately unreliable condition* (σM), the cursor was made up of 25 black dots (⌀ = 1 mm, transparency rate = 40%) with a two-dimensional Gaussian distribution (*SD* = 10 mm), forming a dense cloud. Finally, the *Largely unreliable condition* (σL) had the same characteristics as σM, *but with a standard deviation of 20 mm, resulting in a sparse cloud*. In these last two conditions, only one of the dots in the cloud allowed participants to validate the target, and this reference dot changed every 12 targets (i.e., after every sequence). This reference dot was randomly selected, with no contiguous dots with direct proximity used in succession, and no dot was used twice in a block. For both the σM and σL conditions, five different clouds were used, but with the same distribution characteristics. We created four subgroups per condition (not shown here) to counterbalance the order of the clouds.

The procedure was carried out in three phases, spread over 2 consecutive days (Figure 2). The first day, participants performed the *acquisition phase*. The initial block (i.e., *R*_0_) was the same for all three groups, and featured the *repeated sequence* and the reliable cursor (i.e., σ0 condition). This first block was used to measure the participants’ baseline level and check that it was the same regardless of group. For the remainder of the acquisition phase, participants had their group’s cursor (σ0_,_ σM or σL). Following *R*_0_, a block was presented with the *new sequence* (i.e., *N*_1_). Participants then performed 20 blocks of the *repeated sequence* (i.e., repeated blocks *R*_1_–*R*_20_; see representative trajectories of R1 in Figure 1C). At the 10th block, participants took a 10-min break. Following the completion of the 20 repeated blocks, they performed a second block featuring the *new sequence* (i.e., *N*_2_). Blocks *N*_1_ and *N*_2_ allowed us to measure the sequence-specific learning of the repeated sequence (see Abrahamse et al., 2010, for a review; Boutin et al., 2010, 2014). After approximately 24 h, all the participants returned to perform the *retention phase*, to judge the persistence and transfer of learning. One retention block (featuring the repeated sequence) was performed with the reliable cursor (i.e., retention control, RET_C_), and another with the group cursor (i.e., retention, RET; see representative trajectories of RET in Figure 1C). Their order of presentation was counterbalanced across participants. The RET_C_ block was inserted to compare groups on learning performance. Finally, all the participants performed intermanual transfer tests with the reliable cursor to judge the generalization of learning (Park and Shea, 2005; Kovacs et al., 2009; Boutin et al., 2010, 2012, 2014). More specifically, the transfer tests provided a measure of the extent to which the repeated sequence had been stored and coded. Participants performed a *VisuoSpatial Transfer* block (i.e., TVS) with the *repeated sequence* and the stylus in the *left* hand. They also performed a *Motor Transfer* block (i.e., TM) with the *mirror sequence* (Figure 1B) and the stylus in the *left* hand. The order of presentation was counterbalanced across participants. Figure 2 summarizes the experimental conditions and procedure.

### Data processing

Data processing was performed using MATLAB (version r2020b; Mathworks, Natick, MA). Position data from the tablet were low-pass filtered with a dual-pass, no-lag Butterworth filter (cutoff frequency: 10 Hz; order: 2). These data were used to determine MT per block which corresponded to the mean time (in ms per element) between the validation of the first and 12^th^ elements in the sequence, corresponding to 11 pointing movements. As the path taken to validate the first element in a sequence was not always the same (i.e., at the start of a block, the previous target was the calibration target, whereas for the rest of the block it was the last element of the previous sequence), MT was only calculated for the 11 remaining elements. MT per element was averaged across the block (i.e., for each of the 11 elements across the 10 repeated sequences). Outlier values (median ± 2.5 SD) were removed from the analysis (Leys et al., 2013). These represented 2.2% of the MT per block data. Data analyses were performed with the software JASP (version 0.14.1; JASP, 2020) and consisted of running repeated-measures ANOVAs on MT per block with within factors (i.e., Blocks) and/or between factors (i.e., Groups). The level of significance was set at 0.05 for all statistical analyses, and the effect size was reported for all significant effects (eta-squared, η^2^; Cohen, 1988). When necessary, we applied the Greenhouse and Geisser correction and reported the estimate of sphericity. *Post hoc* tests (Bonferroni) were also performed when necessary. When required, Kruskal–Wallis test was used when the Shapiro–Wilk test rejected the data normality hypothesis.

### Transparency and openness

We reported how we determined our sample size (see “*population*”), all data exclusions, all manipulations, and all measures in the study (see “*data processing*”), and we follow JARS (Kazak, 2018). All data and MATLAB code are available at https://osf.io/xknh4/. This study’s design and its analysis were not pre-registered.

## Results

**Figure 3:**
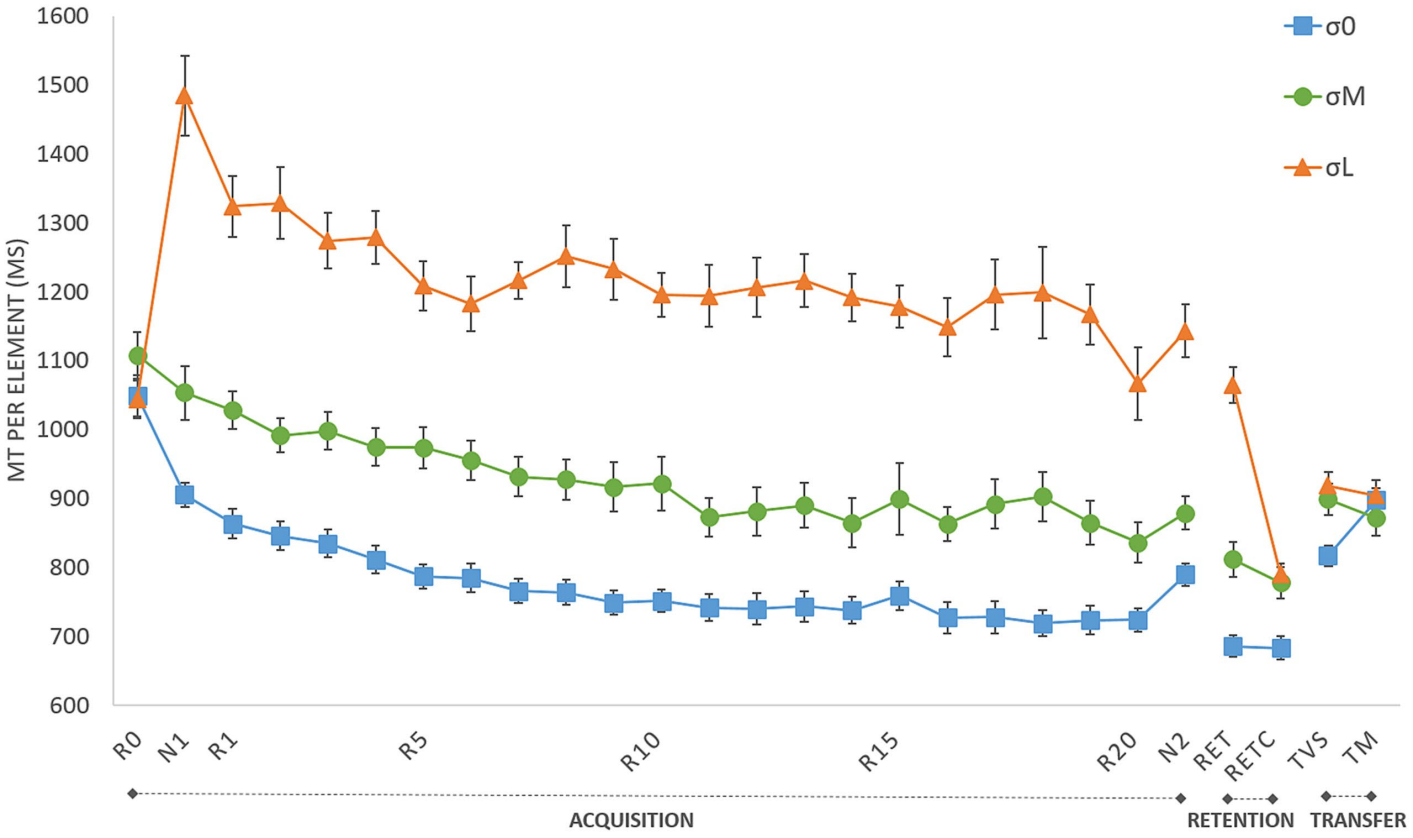
Movement time (MT) per element during acquisition (*R*_0_–*R*_20_: repeated blocks; *N*_1_ and *N*_2_: new blocks), retention (RET: retention with group cursor; RET_C_: retention with control cursor) and transfer tests (TM: motor transfer; TVS: visuospatial transfer). σ0: group with reliable cursor; σM: group with moderately unreliable cursor; σL group with very unreliable cursor. Error bars denote standard error.

Figure 3 depicts MT per block as a function of the three phases of the experiment (i.e., acquisition, retention & transfer) for the three cursor groups (i.e., σ0, σM, σL). The following analyzes characterized (i) the evolution of performances in the acquisition and retention phases; (ii) the sequence-specific learning and (iii) the long-term learning and coding; first for the reliable condition and then for all conditions.

### Learning process in reliable condition

#### Evolution of performances in the acquisition and retention phases

**Figure 4:**
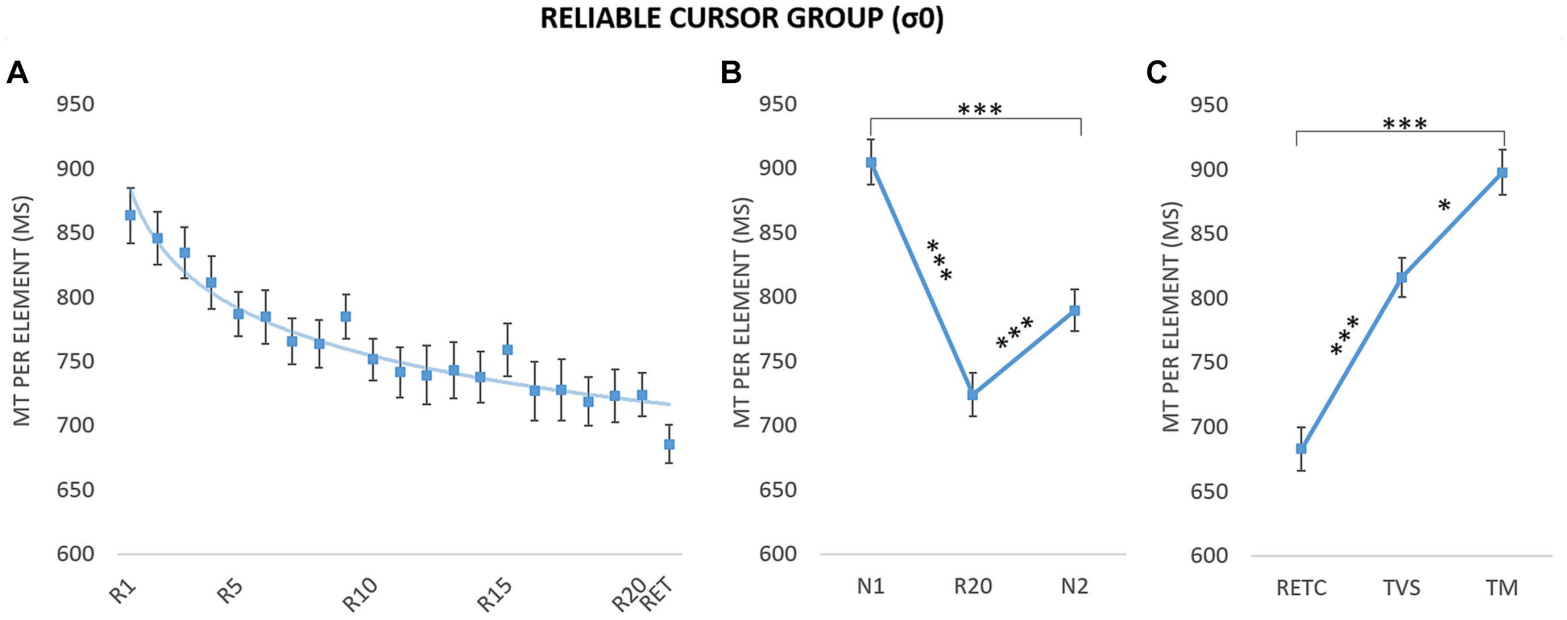
Movement time (MT) per element as a function of block and phase for the group with reliable cursor (σ0). **(A)** Evolution of performance during the acquisition blocks (R1–R20) and retention block (RET). The curve corresponds to the power-law fitted to the mean data: *y* = 882.21x^-0,069^, *R*^2^ = 0.94. **(B)** Sequence-specific learning. *N*_1_ and *N*_2_: new blocks; *R*_20_: last repeated block. **(C)** Retention and transfers. RET_C_: retention with control cursor; TM: motor transfer; TVS: visuospatial transfer. Error bars reflect standard error. *p < 0.05; ***p < 0.001.

It is first important to characterize changes in acquisition performance and learning in the reliable condition. Therefore, we fitted Power-law function on performances to assess the level of skill acquisition (see Newell and Rosenbloom, 1981). The fitting was performed on MT of the reliable condition for the acquisition blocks (R1–R20) and to the retention block (RET, i.e., performed with the same cursor). Participants’ results showed a gradual MT decrement across blocks and a MT maintained in the retention block (RET) with an average intercept equal to *M* = 1003.27 ms (*SE* = 126.76) and to *M* = −0.09 (*SE* = 0.06) for the exponent. Figure 4A illustrates the power-law fitting on the mean data.

### Sequence-specific learning

To assess the learning effect, we used the classic method of including a new sequence N (e.g., Park and Shea, 2005; Boutin et al., 2014) to differentiate between the general improvement (i.e., generalization of practice) and sequence-specific learning (for a review, see Abrahamse et al., 2010). We therefore compared the mean MTs for the last repeated block (*R*_20_) and the pretest (*N*_1_) and posttest (*N*_2_) blocks (Boutin et al., 2014; Figure 4B). A repeated-measures ANOVA revealed a significant main effect of block, *F*(1.73,20.76) = 56.75, *p* < 0.001, η^2^ = 0.83 (estimates of sphericity = 0.86). *Post hoc* tests indicated that R20 was completed faster than N2 (*M* = 723.85 ms, SE = ± 16.92 vs. *M* = 789.47 ms, SE = 16.19 ms; *p* < 0.001), which in turn was completed faster than N1 (*M* = 904.70 ms, SE = 17.60; *p* < 0.001). While the difference between N1 and N2 indicated a generalized practice effect, the difference between R20 and N2 provided a clear indication of sequence-specific learning.

### Long-term learning and movement coding

To assess the persistence of the improvement in performance and to determine the nature of the coding, we compared mean MTs on retention and transfer tests (i.e., visuospatial and motor; Figure 4C). A repeated-measures ANOVA on mean MT for RET_C_, TVS, and TM revealed a main effect of tests, *F*(1.42,17.03) = 62.69, *p* < 0.001, η^2^ = 0.84 (estimates of sphericity = 0.71). RET_C_ was completed faster than TVS (*M* = 682.81 ms, *SE* = 17.00 vs. *M* = 816.14 ms, *SE* = 15.10; *p* < 0.001), which in turn was completed faster than TM (*M* = 897.51 ms, *SE* = 17.53; *p* = 0.02). The difference between TVS and TM indicated visuospatial coding.

### Learning process in unreliable conditions

To understand the differences in learning according to the reliability of the cursor, we compared the performances of the three groups (σ0, σM, and σL).

#### Evolution of performances in the acquisition and retention phase

**Figure 5:**
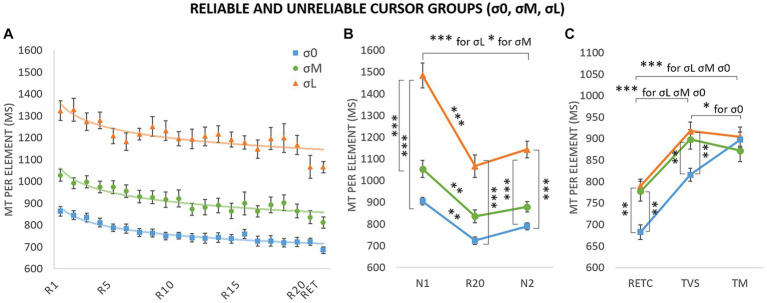
Movement time (MT) per element for the three groups as a function of block and phase. **(A)** Evolution of performance during the acquisition blocks (R1–R20) and retention block (RET). The curve corresponds to the power-law fitted to the mean data; σ0: *y* = 882.21x^–0.069^, *R*^2^ = 0.94; σM: *y* = 1059.30x^–0.068^, *R*^2^ = 0.85; σL: *y* = 1358.00x^–0.056^, *R*^2^ = 0.68. **(B)** Sequence-specific learning. *N*_1_ and *N*_2_: new blocks; *R*_20_: repeated last block. **(C)** Retention and transfer. RET_C_: retention with the reliable control cursor; TM: motor transfer; TVS: visuospatial transfer. σ0: group with reliable cursor; σM: group with moderately unreliable cursor; σL group with very unreliable cursor. Error bars reflect standard error. *p < 0.05; **p < 0.01; ***p < 0.001.

To examine changes in acquisition performance and learning depending on cursor reliability, Power-law functions (see Newell and Rosenbloom, 1981) were fitted to each participant of each groups for the acquisition blocks (R1–R20) and to the retention block (RET, i.e., a block completed with the cursor of the acquisition). Curves’ parameters obtained for each participant (i.e., the intercept and the exponent) were then submitted to statistical analyses. For the intercept, data were submitted to a 3 (group: σ0, σM, σL) ANOVA. First, Shapiro–Wilk analysis indicated that the normality of distribution was respected (ps = 0.94; 0.98; 0.96, respectively) and a Brown-Forsythe correction for homogeneity equal to 0.757 did not change the conclusion. Analysis indicated main effects of group, *F*(2,45) = 27.66, *p* < 0.001, η^2^ = 0.55. *Post hoc* comparisons indicated higher intercept for σL (*M* = 1494.89 ms, *SE* = 251.49) compared to σM (*M* = 1194.69 ms, *SE* = 165.03), itself higher to σ0 (*M* = 1003.27 ms, *SE* = 126.76). For the exponent, data were submitted to a 3 (group: σ0, σM, σL) Kruskal-Wallis test because Shapiro–Wilk analysis indicated that the normality of distribution was not respected. Analysis indicated no main effects of group, *H*(2) = 1.78, *p* = 0.41 (σL: *M* = −0.08, *SE* = 0.07; σM: *M* = −0.11, *SE* = 0.06; σ0: *M* = −0.09, *SE* = 0.06). Altogether, these analyses indicated that participants of each group were progressing at the same rate but with longer MTs for both σL and σM groups and that performances were maintained during the RET test as an index of learning. Figure 5A illustrates the power-law fitting on the mean data of each group.

#### Sequence-specific learning

To assess sequence-specific learning in each of the three groups, we compared MTs in a 3 (group: σ0, σM, σL) × 3 (block: *N*_1_, *R*_20_, *N*_2_) repeated-measures ANOVA (Figure 5B). Analysis revealed main effects of group, *F*(2,32) = 87.90, *p* < 0.001, η^2^ = 0.54, and block, *F*(1.46,46.75) = 46.62, *p* < 0.001, η^2^ = 0.20, and a Block × Group interaction, *F* (2.92,46.75) = 3.00, *p* = 0.04, η^2^ = 0.03 (estimates of sphericity = 0.73). MT was shorter in σ0 than in σM, and shorter in σM than in σL. The differences between σL and σ0 were significant for N1 (*p* < 0.001), R20 (*p* < 0.001), and N2 (*p* < 0.001). The differences between σL and σM were also significant for N1 (*p* < 0.001) and N2 (*p* < 0.001). None of the other intergroup differences were significant. For the σ0 group, the analyzes revealed significant decreases between N1 and R20 (*M* = 904.70 ms, SE = 17.60 vs. *M* = 723.85 ms, SE = 16.92; *p* = 0.002) even if the decrease between N1 and N2 observed in the within-group’s ANOVA (see “Sequence-specific learning analysis” subsection) failed to reach significance (N2 mean: *M* = 789.47 ms, SE = 16.19; *p* = 0.40); as well as the increase between R20 and N2 (*p* = 1.00). Likewise, for σM, we observed decreases between N1 and R20 (*M* = 1052.99 ms, SE = 39.26 vs. *M* = 836.25 ms, SE = 22.84; *p* = 0.01) and between N1 and N2 (*M* = 879.32, SE = 23.07; *p* = 0.02) but no significant difference between R20 and N2 (*p* = 1.00). The same overall pattern was found for σL, with decreases between N1 and R20 (*M* = 1484.02 ms, SE = 57.87 vs. *M* = 1066.61 ms, SE = 40.03; *p* < 0.001) and between N1 and N2 (*M* = 1143.33 ms, SE = 38.77; *p* < 0.001), but no significant difference between R20 and N2 (*p* = 1.00). Analysis revealed a generalized practice effect for unreliable groups (N1 vs. N2), but no sequence-specific learning (R20 vs. N2). The variability introduced by the unreliable groups probably absorbed the significant differences for σ0 in the ANOVA’s comparisons.

#### Long-term learning and movement coding

To assess group differences on transfer, we conducted a 3 (group: σ0, σM, σL) × 3 (block: RET_C_, TVS, TM) repeated-measures ANOVA (Figure 5C). The retention block was introduced to compare the efficiency of coding versus optimum performance (Park and Shea, 2005; Kovacs et al., 2009; Boutin et al., 2010, 2014). Analysis indicated main effects of group, *F*(2,33) = 9.34, *p* < 0.001, η^2^ = 0.15, and block *F*(1.72,56.59) = 120.00, *p* < 0.001, η^2^ = 0.44, as well as a group x block interaction, *F*(3.43,56.59) = 4.49, *p* = 0.003, η^2^ = 0.03 (estimates of sphericity = 0.86). MT was significantly longer for TVS (σL: *M* = 917.65 ms, *SE* = 20.82; σM: *M* = 898.35 ms, *SE* = 22.75; σ0: *M* = 816.14 ms, *SE* = 15.10) than for RET_C_ (*M* = 789.83 ms, *SE* = 16.13; *M* = 777.70 ms, *SE* = 23.18; *M* = 682.81 ms, *SE* = 17.00; *p* < 0.001), and significantly longer for TM than for RET_C_ (*M* = 896.09 ms, *SE* = 22.47; *M* = 871.85 ms, *SE* = 25.55; *M* = 897.51 ms, *SE* = 17.53; *p* < 0.001). MT was shorter for σ0 than for either σM (*p* = 0.01) and σL (*p* = 0.002) for RET_C_ and for σM (*p* = 0.01) and σL (*p* = 0.01) for TVS. Analysis did not reveal any significant intergroup difference for TM (*p* = 1.00). Lastly, we found a longer MT for TM, compared with TVS, for σ0 (*p* = 0.01). No such difference was found for the other two groups (*p* = 1.00).

## Discussion

Our main objective was to assess the effect of visual cursor reliability on the learning and movement coding of a continuous motor task. First, our analyses revealed an improvement in performance for the reliable group (σ0) that could not result solely from visuohaptic calibration (i.e., set up and task familiarization): MT was shorter for the repeated sequence than for the new sequence in the posttest (i.e., *R*_20_ vs. N_2_ comparison). This typical learning process for the reliable condition was confirmed by a gradual decrease in MT during acquisition (i.e., power-law fitting). By contrast, our results showed that unreliable visual cursor disrupts the long-term learning of a sequence, for although we observed retention of performance for σL after 24 h (i.e., *R*_0_ vs. RET_C_), MT remained longer than for σ0. Finally, analyses of transfer yielded the classic finding of better performance on TVS than on TM for σ0, but no such difference was observed when the cursor was moderately (σM) or very (σL) unreliable. Interestingly, these results indicated for the first time that the type of coding of the sequence depends on the reliability of the cursor.

In line with previous results (e.g., Park and Shea, 2005; Blandin et al., 2008; Kovacs et al., 2009; Boutin et al., 2010, 2013a, 2014), we expected to observe a gradual decrease in MT across the acquisition phase. Here, we observed an overall decrease in MT across the blocks, whatever the reliability of the cursor (see power-law fittings). MTs were longer for σM and longer still for σL, compared with the reliable group (σ0), owing to the noise generated by the search for the reference dot, which particularly disturbed participants’ movements at the beginning of practice (see representative trajectories Figure 1C). Conversely, their MT decreased with practice. To assess the learning effect, we used the classic method of including a control block (e.g., Park and Shea, 2005; Boutin et al., 2014) to differentiate between the general improvement (i.e., generalization of practice) and *sequence-specific learning* (for a review, see Abrahamse et al., 2010). In the reliable group (σ0), in accordance with Boutin et al. (2010, 2013b, 2014), we observed a higher MT for *N*_1_ relative to *N*_2_ and a higher MT for *N*_2_ relative to *R*_20_, indicative of sequence-specific learning, but no such decrease was observed in the unreliable cursor’s groups. To our knowledge, this is the first time that unreliable visual cursor has been shown to lead to a decrease in learning performance on a sequential task. As visual information was available throughout the experiment, performance remained optimum for σ0, which may even have developed visual dependency (Blandin et al., 2008). By contrast, when the cursor was unreliable during acquisition (i.e., for σL and σM), participants performed more poorly than controls (σ0) on retention tests, even when there was a reliable cursor (i.e., RET_C_). We hypothesized that participants in σL and σM learned to rely more on other sensory sources (e.g., proprioceptive cues), as these were more reliable during acquisition (Körding and Wolpert, 2004; Berniker and Kording, 2008; Hewitson et al., 2018), and they continued to use these sources even when they could benefit from reliable on-line visual feedback. One may argue that this reweighting toward other sensory sources may also be linked to the change of the visuomotor mapping during σL and σM conditions. Indeed, the cloud of dots may not only alter the reliability of the visual cursor, but also modify the visuomotor mapping between the hand and visual cursor positions. Participants would have been required to learn the stochastic visuomotor perturbation as the reference dot which validated the target had changed every 12 targets. Adaptation to visuomotor perturbations was shown to lead to slower and less complete learning when the standard deviation of the visuomotor rotations distribution was higher (Fernandes et al., 2012).

To observe the coding process, it is common to perform delayed visuospatial and motor transfer tests (e.g., Kovacs et al., 2009). For TVS, participants had to produce the same sequence as during acquisition or retention (i.e., repeated sequence), but with the contralateral hand: the visuospatial pattern was thus maintained, but the pattern of muscle activation and the joint angles were opposed. For TM, participants had to produce a mirror sequence using the contralateral hand: the pattern of muscle flexion and extension was thus the same as during the repeated blocks, but the visuospatial pattern was mirrored. For the group with reliable cursor (i.e., σ0), we confirmed the results of previous studies reporting longer MTs for TM than for TVS (e.g., Kovacs et al., 2009; Boutin et al., 2010, 2012): at this stage of learning, visuospatial coding was therefore more developed than motor coding. Furthermore, we first showed a link between cursor reliability and movement coding. For groups with unreliable cursor (i.e., σM and σL), we observed similar MTs for both TVS and TM. Thus, when participants initiated visuospatial coding, they also initiated motor coding, unlike those in the reliable group. This link was supported by the computation of the deterioration rates for TM ([MT of TM – MT of RET_C_]/MT of RET_C_) and for TVS ([MT of TVS – MT of RET_C_]/MT of RET_C_). Whereas σL and σM had similar deterioration rates for both TM (0.14 for σL and 0.13 for σM) and TVS (0.16 for both groups), σ0 had a greater deterioration rate for TM (0.31) than for TVS (0.19).

The nature of movement coding depends on task’s context and individual’s strategy (Blouin et al., 2014), as well as practice’s conditions (Hikosaka et al., 1999, 2002) suggesting a binary process. This means that if visuospatial coding was the dominant type at the start of the acquisition phase, motor coding could only replace it as a result of practice. However, Kovacs et al. (2009) found that TVS remained better than TM regardless of the amount of practice (i.e., 1, 4, or 12 days). To the best of our knowledge, our results indicated, for the first time, that the reliability of sensory information during training influenced the type of coding that was implemented: the more available and reliable the visual information during acquisition, the greater the implementation of visuospatial coding. By contrast, when this cursor was degraded, participants relied more on other available sources of sensory information, and engaged in both visuospatial and motor coding. This mixed coding showed that, depending on the context, one or other type of coding could be activated (Kovacs et al., 2009). One cannot exclude that this mixed coding hypothesis could be rather a unique coding/reference frame which would include more or less weight to the visual and proprioceptive cues reliable in this pointing task. The resulting multisensory coding would obey the “reliability rule” (Colonius and Diederich, 2020): the weights in averaging the unisensory estimates would be proportional to their relative reliabilities. Several studies showed that the perceived positions of the hand (conveyed by proprioception) and the object to reach (conveyed by vision) are biased toward each other (e.g., Rand and Heuer, 2013; Debats et al., 2017). This perceptual attraction has been shown to be modulated by the reliability of the hand position and the visual object position reliability (Debats et al., 2017). The brain would combine sensory sources in an optimal fashion providing an amodal unique representation/coding (Kirsch et al., 2016).

## Conclusion

Overall, this study assessed the impact of the reliability of on-line visual feedback on sequence learning and how the motor sequence was coded. Although unreliable visual cursor had a negative impact on retention, it also stimulated mixed coding (visuospatial plus motor coding), promoting better sequence learning. As mentioned by Wulf et al. (2010), the most beneficial conditions for learning in the short term (i.e., acquisition phase) are not necessarily the most beneficial in the long term for effective learning as retained or generalizable skill or knowledge. Therefore, here the beneficial impact of visual unreliability on motor coding constitutes an interesting output, notably in the domain of education, performance or reeducation. Interesting perspectives also appear regarding the manipulation of the visual feedback reliability over several consecutive days of practice as Kovacs et al. (2009) showed that coding evolves over short to long term practice (1–14 days). In addition, here the reliability of the visual feedback was only manipulated at the task level and modulating contextual interference *during* the learning of a task (e.g., by manipulating the practice conditions) may facilitate even more the long-term learning process (for a review, see Wright et al., 2016). Future studies should manipulate reliability *during* practice in a within participants design.

## Data availability statement

The datasets presented in this study can be found in online repositories. The names of the repository/repositories and accession number(s) can be found at: https://osf.io/xknh4/.

## Ethics statement

The studies involving humans were approved by General data protection regulations (GDPR) of the University of Poitiers. The studies were conducted in accordance with the local legislation and institutional requirements. The participants provided their written informed consent to participate in this study.

## Author contributions

CS and YB conceived and designed the research. GC, MB, and CS set up the experiment. MB performed the experiments. CS, MB, and YB analyzed the data. CS, YB, and MB interpreted the results of the experiments. MB and CS prepared the figures. MB, CS, and YB drafted the manuscript. CS, YB, GC, and MB edited and revised the manuscript. All authors contributed to the article and approved the submitted version.

## References

[ref1] Abrahamse E. L. Jiménez L. Verwey W. B. Clegg B. A. (2010). Representing serial action and perception. Psychon. Bull. Rev. 17, 603–623. doi: 10.3758/PBR.17.5.603, PMID: 21037157

[ref2] Berniker M. Kording K. (2008). Estimating the sources of motor errors for adaptation and generalization. Nat. Neurosci. 11, 1454–1461. doi: 10.1038/nn.2229, PMID: 19011624 PMC2707921

[ref3] Blandin Y. Toussaint L. Shea C. H. (2008). Specificity of practice: interaction between concurrent sensory information and terminal feedback. J. Exp. Psychol. Learn. Mem. Cogn. 34, 994–1000. doi: 10.1037/0278-7393.34.4.994, PMID: 18605884

[ref4] Blouin J. Saradjian A. H. Lebar N. Guillaume A. Mouchnino L. (2014). Opposed optimal strategies of weighting somatosensory inputs for planning reaching movements toward visual and proprioceptive targets. J. Neurophysiol. 112, 2290–2301. doi: 10.1152/jn.00857.2013, PMID: 25122716

[ref5] Boutin A. Badets A. Salesse R. N. Fries U. Panzer S. Blandin Y. (2012). Practice makes transfer of motor skills imperfect. Psychol. Res. 76, 611–625. doi: 10.1007/s00426-011-0355-2, PMID: 21671102

[ref6] Boutin A. Blandin Y. (2010). Cognitive underpinnings of contextual interference during motor learning. Acta Psychol. 135, 233–239. doi: 10.1016/j.actpsy.2010.07.004, PMID: 20684941

[ref7] Boutin A. Blandin Y. Massen C. Heuer H. Badets A. (2014). Conscious awareness of action potentiates sensorimotor learning. Cognition 133, 1–9. doi: 10.1016/j.cognition.2014.05.012, PMID: 24954450

[ref8] Boutin A. Fries U. Panzer S. Shea C. H. Blandin Y. (2010). Role of action observation and action in sequence learning and coding. Acta Psychol. 135, 240–251. doi: 10.1016/j.actpsy.2010.07.005, PMID: 20673569

[ref9] Boutin A. Massen C. Heuer H. (2013a). Modality-specific organization in the representation of sensorimotor sequences. Front. Psychol. 4:937. doi: 10.3389/fpsyg.2013.00937, PMID: 24376432 PMC3858678

[ref10] Boutin A. Panzer S. Blandin Y. (2013b). Retrieval practice in motor learning. Hum. Mov. Sci. 32, 1201–1213. doi: 10.1016/j.humov.2012.10.002 24060222

[ref14] Coello Y. Orliaguet J. P. Prablanc C. (1996). Pointing movement in an artificial perturbing inertial field: a prospective paradigm for motor control study. Neuropsychologia 34, 879–892. doi: 10.1016/0028-3932(96)00003-6, PMID: 8822735

[ref15] Cohen J., (1988). Statistical power analysis for the behavioral sciences, 2nd Ed. L. Erlbaum Associates: Hillsdale, NJ.

[ref16] Colonius H. Diederich A. (2020). Formal models and quantitative measures of multisensory integration: a selective overview. Eur. J. Neurosci. 51, 1161–1178. doi: 10.1111/ejn.13813, PMID: 29285815

[ref17] Criscimagna-Hemminger S. E. Donchin O. Gazzaniga M. S. Shadmehr R. (2003). Learned dynamics of reaching movements generalize from dominant to nondominant arm. J. Neurophysiol. 89, 168–176. doi: 10.1152/jn.00622.2002, PMID: 12522169

[ref18] Debats N. B. Ernst M. O. Heuer H. (2017). Perceptual attraction in tool use: evidence for a reliability-based weighting mechanism. J. Neurophysiol. 117, 1569–1580. doi: 10.1152/jn.00724.2016, PMID: 28100656 PMC5376609

[ref20] Ernst M. O. Banks M. S. (2002). Humans integrate visual and haptic information in a statistically optimal fashion. Nature 415, 429–433. doi: 10.1038/415429a, PMID: 11807554

[ref21] Faul F. Erdfelder E. Lang A.-G. Buchner A. (2007). G*power 3: a flexible statistical power analysis program for the social, behavioral, and biomedical sciences. Behav. Res. Methods 39, 175–191. doi: 10.3758/BF03193146, PMID: 17695343

[ref22] Fernandes H. L. Stevenson I. H. Kording K. P. (2012). Generalization of stochastic visuomotor rotations. PLoS One 7:e43016. doi: 10.1371/journal.pone.0043016, PMID: 22916198 PMC3419239

[ref23] Hewitson C. L. Sowman P. F. Kaplan D. M. (2018). Interlimb generalization of learned Bayesian Visuomotor prior occurs in extrinsic coordinates. eNeuro 5:ENEURO.0183-18.2018. doi: 10.1523/ENEURO.0183-18.2018, PMID: 30131969 PMC6102376

[ref24] Hikosaka O. Nakahara H. Rand M. K. Sakai K. Lu X. Nakamura K.. (1999). Parallel neural networks for learning sequential procedures. Trends Neurosci. 22, 464–471. doi: 10.1016/s0166-2236(99)01439-3 10481194

[ref25] Hikosaka O. Nakamura K. Sakai K. Nakahara H. (2002). Central mechanisms of motor skill learning. Curr. Opin. Neurobiol. 12, 217–222. doi: 10.1016/s0959-4388(02)00307-0 12015240

[ref01] Kazak A. E. (2018). Editorial: Journal article reporting standards. Am. Psychol. 73, 1–2. doi: 10.1037/amp0000263 29345483

[ref26] Kirsch W. Pfister R. Kunde W. (2016). Spatial action-effect binding. Atten. Percept. Psychophys. 78, 133–142. doi: 10.3758/s13414-015-0997-z, PMID: 26486641

[ref27] Körding K. P. Wolpert D. M. (2004). Bayesian integration in sensorimotor learning. Nature 427, 244–247. doi: 10.1038/nature02169 14724638

[ref28] Kovacs A. J. Mühlbauer T. Shea C. H. (2009). The coding and effector transfer of movement sequences. J. Exp. Psychol. Hum. Percept. Perform. 35, 390–407. doi: 10.1037/a0012733, PMID: 19331496

[ref29] Lange R. K. Godde B. Braun C. (2004). EEG correlates of coordinate processing during intermanual transfer. Exp. Brain Res. 159, 161–171. doi: 10.1007/s00221-004-1942-x, PMID: 15340766

[ref30] Leys C. Ley C. Klein O. Bernard P. Licata L. (2013). Detecting outliers: do not use standard deviation around the mean, use absolute deviation around the median. J. Exp. Soc. Psychol. 49, 764–766. doi: 10.1016/j.jesp.2013.03.013

[ref32] Newell A. Rosenbloom P. (1981). “Mechanisms of skill acquisition and the law of practice,” in Cognitive skills and their acquisition. ed. Anderson J. R. (Hillsdale: Erlbaum), 1–55.

[ref34] Park J.-H. Shea C. H. (2002). Effector independence. J. Mot. Behav. 34, 253–270. doi: 10.1080/00222890209601944 19260176

[ref35] Park J.-H. Shea C. H. (2005). Sequence learning: response structure and effector transfer. Q. J. Exp. Psychol. A 58, 387–419. doi: 10.1080/02724980343000918, PMID: 16025755

[ref36] Proteau L. (2005). Visual afferent information dominates other sources of afferent information during mixed practice of a video-aiming task. Exp. Brain Res. 161, 441–456. doi: 10.1007/s00221-004-2090-z, PMID: 15517215

[ref37] Rand M. K. Heuer H. (2013). Implicit and explicit representations of hand position in tool use. PLoS One 8:e68471. doi: 10.1371/journal.pone.0068471, PMID: 23894307 PMC3716878

[ref38] Robin C. Toussaint L. Blandin Y. Vinter A. (2004). Sensory integration in the learning of aiming toward “self-defined” targets. Res. Q. Exerc. Sport 75, 381–387. doi: 10.1080/02701367.2004.10609171, PMID: 15673037

[ref39] Sato Y. Kording K. P. (2014). How much to trust the senses: likelihood learning. J. Vis. 14:13. doi: 10.1167/14.13.13, PMID: 25398975 PMC4233767

[ref40] Sigrist R. Rauter G. Riener R. Wolf P. (2013). Augmented visual, auditory, haptic, and multimodal feedback in motor learning: a review. Psychon. Bull. Rev. 20, 21–53. doi: 10.3758/s13423-012-0333-8, PMID: 23132605

[ref42] Tremblay L. Proteau L. (1998). Specificity of practice: the case of powerlifting. Res. Q. Exerc. Sport 69, 284–289. doi: 10.1080/02701367.1998.10607695, PMID: 9777665

[ref44] Wolpert D. M. Diedrichsen J. Flanagan J. R. (2011). Principles of sensorimotor learning. Nat. Rev. Neurosci. 12, 739–751. doi: 10.1038/nrn3112 22033537

[ref45] Wright D. Verwey W. Buchanen J. Chen J. Rhee J. Immink M. (2016). Consolidating behavioral and neurophysiologic findings to explain the influence of contextual interference during motor sequence learning. Psychon. Bull. Rev. 23, 1–21. doi: 10.3758/s13423-015-0887-3, PMID: 26084879

[ref46] Wulf G. Shea C. Lewthwaite R. (2010). Motor skill learning and performance: a review of influential factors. Med. Educ. 44, 75–84. doi: 10.1111/j.1365-2923.2009.03421.x, PMID: 20078758

